# Supplementary material for: High-frequency head impact causes chronic synaptic adaptation and long-term cognitive impairment in mice
Source: Nat Commun. 2021 May 10;12:2613. doi: 10.1038/s41467-021-22744-6 (PMC8110563; doi:10.1038/s41467-021-22744-6)

High-frequency head impact causes chronic synaptic adaptation and long-term cognitive impairment in mice

Stephanie S. Sloley,§ Bevan S. Main,§ Charisse N. Winston, Alex C. Harvey, Alice Kaganovich, Holly T. Korthas, Adam P. Caccavano, David N. Zapple, Jian-young Wu, John G. Partridge,$ Mark R. Cookson,$ Stefano Vicini,$ and Mark P. Burns$

§, $ = contributed equally

SUPPLEMENTAL INFORMATION

Supplemental Methods:

Western Blot

Western blot analysis performed as previously described.[1] Briefly, tissue was homogenized in RIPA buffer (Pierce, #89900) containing protease and phosphatase inhibitors (Pierce, #88668). 20µg of protein was added to Laemmli sample buffer (1610737, BioRad, Hercules, CA) containing 5% v/v β-mercaptoethanol (1610710, BioRad) and heated to 95°C for 5 minutes. Proteins were separated via SDS-PAGE gel electrophoresis, before transfer to nitrocellulose membranes. Primary antibodies (Key Resources) were added to 2% w/v skim milk/TBS-T and applied to membranes for overnight incubation at 4°C. HRP-conjugated goat anti-rabbit or goat anti-mouse secondary antibodies (Jackson Immunoresearch, Westgrove, PA, 1:1000 in 2% w/v skim milk powder in PBS-T) were used before detection with SuperSignal West Pico Chemmiluminescent Substrate (34080, Thermoscientific, Waltham, MA). Visualization of chemiluminescent signal was done with Amersham 600 imaging machine (GE healthcare, Chicago, IL). Raw pixel intensity of bands from the blots were quantified using Image J software densitometry plugins (Version 1.47, NIH, Bethesda, MD). Full scans of key blots are available in the source data file.

Immunohistochemistry and quantitative analysis.

4% paraformaldehyde fixed brain slices (20 µm) sections were incubated in 0.3% H_2O_2 and blocked with 3% normal goat serum in PBS with 0.25% Triton X-100 before incubation with primary antibodies (see table below) in PBS, 0.25% Triton X-100 and 1% normal goat serum. Biotinylated goat anti-rabbit or anti-mouse secondary antibodies (1:2000, Vector laboratories, Burlingame, CA; catalog number BA-100) in a 0.25% Triton X-100 and PBS solution followed by Vectastain avidin/biotinylated enzyme complex kit (1:400, Vector Laboratories; catalog number PK-6100) and a 3, 3'-diaminobenzidine (Sigma-Aldrich, St. Louis, MO) to visualize the primary antibodies.

For quantitative analysis of immunolabeled sections, we implemented unbiased standardized

sampling techniques to measure tissue areas corresponding to the hippocampus, corpus callosum, and optic tract as previously described.[2] To quantify the number and area of Iba-1 and CD68 positive cells, an average of five single plane sections from the lesion epicenter (–1.34 to – 2.30 mm from bregma) were analyzed for each animal for the hippocampus and corpus callosum, and three single plane sections for optic tract. Data is presented as the mean number of cells per mm^2. For proportional area measurements, the magnitude of the individual reaction for microglial and astroglial cells was reported as the proportional area of tissue occupied by immunohistochemical stained cellular profiles within a defined target area. Microscope images were transferred to ImageJ software, for inversion and thresholding, and densitometric analysis was performed as previously described.[3,4] The thresholding function is used to set a black and white threshold corresponding to the imaged field, with the averaged background subtracted out. Once a threshold is set, the "Analyze Particles" function can be used to sum up the total area of positive staining, and to calculate the fraction of the total area that is positive for the stain. Data are shown as the percentage of Iba-1 or CD68 positive immunoreactivity per the total area studied.

ELISA

$A\beta_{40}$ measurements were conducted on cortical and hippocampal brain regions as previously described.[5,6] Brain regions were homogenized in 0.4% diethylamine (sigma
#471216) in a 250mM sucrose, 20mM Tris, 1mM EDTA and 1mMEGTA buffer. Concentration of endogenous mouse $A\beta_{40}$ were detected using an ELISA (Invitrogen #KMB3481). For cytokine assessment, cortical and hippocampal brain regions were isolated before homogenization in RIPA buffer (Pierce, #89900) containing protease and phosphatase inhibitors (Pierce, #88668). IL-1β (R&D Systems, #DY401) and TNFα (R&D Systems, #DY410) cytokine ELISAs were performed according to manufacturer's instructions.

Supplementary Methods Table 1. List of TAQMAN gene expression assays used in RT-QPCR

Gene Name	Product Number	RefSeq	Amplicon length (bp)
Arc	Mm00479619_g1	NM_018790.3	71
Shank2	Mm01163731_m1	NM_001081370.2	71
Ppp3r1	Mm01187904_m1	NM_024459.2	97
Shank3	Mm00498775_m1	NM_021423.3	76
Dlgap4	Mm00525149_m1	NM_001042487.1	87
Gapdh	Mm99999915_g1	NM_001289726.1	107
Ubc	Mm02525934_g1	NM_019639.4	176
Rn18s	Mm03928990_g1	NR_003278.3	61
Bsn	Mm00464452_m1	NM_007567.2	74
Cacnb3	Mm00432244_m1	NM_001044741.2	119
Camk2a	Mm00437967_m1	NM_001286809.1	83
Brsk1	Mm01328475_m1	NM_001003920.3	57
Ppp1r9b	Mm00552071_m1	NM_172261.3	107
Slc17a1	Mm00436577_m1	NM_001170638.1	113
Slc17a7	Mm00812886_m1	NM_182993.2	55
Camta2	Mm00626346_m1	NM_001190376.1	75

Supplementary Methods Table 2. List of antibodies used for Western and immunohistochemistry

Antibody	Supplier	Secondary	Dilution
Phospho-Tau (Ser199, Ser202)	Invitrogen (44-768G)	Anti-Rabbit HRP	1:1000
Phospho-Tau (Ser396)(PHF13)	Cell Signaling (9632)	Anti-Mouse HRP	1:1000
Phospho-Tau (Ser416)(D7U2P)	Cell Signaling (15013)	Anti-Rabbit HRP	1:1000
β-Actin	Sigma-Aldrich (A5441)	Anti-Mouse HRP	1:5000
Amyloid precursor protein (APP)	Invitrogen (36-6900)	Anti-Rabbit HRP	1:1000
Iba1	Wako (019-19741)	Anti-Rabbit HRP	1:1000
CD68	Serotec (MCA1957T)	Anti-mouse HRP	1:1000

Supplementary Methods Table 3- Electrophysiology solutions

Name	Components (mM)
Sucrose cutting solution	containing: 3 KCl, 1.25 NaH_2PO_4, 26 $NaHCO_3$, 2 $MgSO_4$, 2 $CaCl_2$, 10 dextrose and 252 sucrose.
Artificial Cerebrospinal Fluid (aCSF)	132 NaCl, 26 $NaHCO_3$, 3 KCl, 1.25 NaH2PO_4, 2 $MgSO_4$, 2 $CaCl_2$ and 10 dextrose) with constant saturation in 95% O_2 and 5% CO_2.
N-methyl D-glucamine (NMDG) solution	93 NMDG, 2.5 KCl, 1.2 NaH_2PO_4, 30 $NaHCO_3$, 20 HEPES, 25 dextrose, 10 sucrose, 5 ascorbic acid, 2 thiourea, 3 sodium pyruvate, 5 N-acetyl-L-cysteine (NAC), 10 $MgSO_4$ 7H_2O, and 0.5 $CaCl_2$ 2H_2O. The solution was brought to a pH of 7.3- 7.4, and an osmolarity of 300-310 mOsM, and was bubbled with 95% O_2 and 5% CO_2 30 minutes prior to perfusion.
Incubation solution	92 NaCl, 2.5 KCl, 1.2 NaH_2PO_4, 30 $NaHCO_3$, 20 HEPES, 25 dextrose, 5 ascorbic acid, 2 thiourea, 3 sodium pyruvate 5 NAC, 2 $MgSO_4$ 7H_2O, and 2 $CaCl_2$ 2H_2O. The solution had a pH of 7.3-7.4 and an osmolarity of 300-310 mOsm/kg, and was constantly bubbled with 95% O_2 and 5% CO_2.
aCSF2	124 NaCl, 4.5 KCl, 1 $MgCl_2$ 2H_2O, 1.2 NaH_2PO_4, 10 dextrose, 26 $NaHCO_3$, and 2 $CaCl_2$ (pH 7.4, bubbled constantly with 95% O_2 and 5% CO_2).
potassium gluconate (kgluc) internal solution	145 potassium gluconate, 10 HEPES, 5 EGTA, 5 ATP·Mg 0.2 GTP·Na, osmolarity 305 mOsm, adjusted to pH 7.20.
Cesium Methanesulfonate (CsMe) internal solution	120 cesium methanesulfonate, 5 NaCl, 10 tetraethylammonium chloride, 10 HEPES, 4 QX314, 1.1 EGTA, 5 MgATP, and 0.2 NaGTP, osmolarity 305 mOsm, with a pH adjusted to 7.25 using CsOH.

Supplemental Method References

1 Main, B. S. *et al.* Apolipoprotein E4 impairs spontaneous blood brain barrier repair following traumatic brain injury. *Molecular neurodegeneration* **13**, 17, doi:10.1186/s13024-018-0249-5 (2018).

2 Villapol, S., Loane, D. J. & Burns, M. P. Sexual dimorphism in the inflammatory response to traumatic brain injury. *Glia* **65**, 1423-1438, doi:10.1002/glia.23171 (2017).

3 Villapol, S., Balarezo, M. G., Affram, K., Saavedra, J. M. & Symes, A. J. Neurorestoration after traumatic brain injury through angiotensin II receptor blockage. *Brain* **138**, 3299-3315, doi:10.1093/brain/awv172 (2015).

4 Villapol, S., Byrnes, K. R. & Symes, A. J. Temporal dynamics of cerebral blood flow, cortical damage, apoptosis, astrocyte-vasculature interaction and astrogliosis in the pericontusional region after traumatic brain injury. *Front Neurol* **5**, 82, doi:10.3389/fneur.2014.00082 (2014).

5 Washington, P. M., Morffy, N., Parsadanian, M., Zapple, D. N. & Burns, M. P. Experimental traumatic brain injury induces rapid aggregation and oligomerization of amyloid-beta in an Alzheimer's disease mouse model. *J Neurotrauma* **31**, 125-134, doi:10.1089/neu.2013.3017 (2014).

6 Loane, D. J. *et al.* Amyloid precursor protein secretases as therapeutic targets for traumatic brain injury. *Nat.Med.* **15**, 377-379 (2009).

Supplemental Figure 1. Cortical transcriptomic signature 24h after HF-HI.

(A) Schematic outlining RNA-seq analysis of cortical responses following HF-HI procedure. **(B)** Heatmap showing levels of all significantly altered genes ($p < 0.05$) in cortex as identified by RNA-seq. Color scale bar values represent Z-value (normalized standard deviations from the mean) for expression relative to the overall mean expression. **(C)** Functional enrichment of differentially expressed genes in HF-HI mice using gene sets from gene ontology (GO) displaying cellular components (CC) and biological processes (BP). **(D)** Heat map displaying differentiated genes in neuron part (CC) and glutamatergic synapse KEGG categories. Each gene is colored by Z-value for expression relative to the overall mean expression for that gene. Individual samples listed below each heat map. **(E)** RT-QPCR validation of key synaptic signaling genes in the cortex. Data shown as mean +/- SEM**.**, Sham n=10, HF-HI n=11; unpaired, two-tailed t-test. **(F)** RT-QPCR quantification of key synaptic genes in the hippocampus. Data shown as mean +/- SEM**.** Sham n=10, HF-HI n=11; unpaired, two-tailed t-test.

Supplemental Figure 2. Transcriptomic network analysis 24h after HF-HI.

Map of significantly represented pathways altered by HF-HI, generated using the enrichment map Cytoscape plug-in. Network analysis performed using false discovery rate (FDR) correction. BP, CC and KEGG networks are depicted for each gene set. Node size corresponds to the number of genes in each ontology term. Only terms ranging between 10 and 3000 were included in this analysis. Node color corresponds to which gene set each enrichment is derived from. Edges signify significant overlap between terms using a cutoff of 0.5 similarity coefficient, darker edges indicate greater overlap between terms.

Supplemental Figure 3. HF-HI does not induce neuroinflammation at 24h or 1m post injury

(A) Immunohistochemical analysis of Iba1 and CD68 in sham and HF-HI mouse brains at 1d an 1m post-impact. Iba1 and CD68 staining showed no evidence of changes to microglia/macrophage number or morphology in the cortex, hippocampus or corpus callosum at either timepoint. There was

a strong accumulation of both Iba1 and CD68 positive microglia/macrophages in the optic tract in the optic tract of HF-HI mice at both timepoints (sham 24h n = 4, HF-HI 24h n= 8, sham 1m n = 4, HF-HI 1m n = 5). **(B-C)** Region specific protein analysis of inflammatory cytokines IL1-β and TNF-α reveals no difference between sham and HF-HI groups in cortex or hippocampus at 24h (n = 4) or 1m post injury (n = 6). **(D)** RT-QPCR analysis shows no change in cortical Iba1 (*Aif1*) or *Gfap* mRNA expression between sham and HF-HI mice at 1m post injury (n = 6 per group). Data shown as mean +/- SEM**.**

Supplemental Figure 4. HF-HI does not cause accumulation of APP, phosphorylated tau or Aβ

(A-B) Representative Western blots and densitometry for APP and p-tau epitopes Ser199/202, Ser416, and Ser396 in C57Bl/6 mouse cortex (Ctx) and hippocampus (Hpc) at 24h post HF-HI. P-tau signal was normalized to β-actin. HF-HI caused no change in endogenous tau phosphorylation at any of the quantified epitopes (Sham n=7, HF-HI n=8). Quantification of $A\beta_{40}$ from DEA-soluble cortical and hippocampal brain homogenates at **(C)** 1d and **(D)** 1m show that HF-HI had no effect on $A\beta_{40}$ accumulation. (1d Sham n=7, HF-HI n=8, 1m n=6/group). **(E-F)** Representative Western blots and densitometry for APP and p-tau epitopes Ser199/202, Ser416, and Ser396 in C57Bl/6 mouse cortex (Ctx) and hippocampus (Hpc) at 1m post HF-HI. P-tau signal was normalized to β-actin. HF-HI caused no change in endogenous tau phosphorylation at any of the quantified epitopes (n=6/group). Data shown as mean +/- SEM**.**

Supplemental Figure 5. HFHI does not alter spine density in multiple brain regions, or the percent charge transfer of CA1 neurons.

(A) Dendritic spine analysis from the apical oblique dendrites of Layer II/III cortical neurons show that there was no effect of HF-HI on spine density. Data shown as the number of spines per 20µm neuron segment (n = 182 sham and 184 HF-HI neurons from 15 mice per group). **(B)** Dendritic spine analysis from the basal shaft dendrites of Layer II/III cortical neurons show that there was no effect of HF-HI on

spine density. Data shown as the number of spines per 20μm neuron segment (n = 60 sham and 58 HF-HI neurons from 5 mice per group). **(C)** Dendritic spine analysis from the dentate gyrus hippocampal neurons show that there was no effect of HF-HI on spine density. Data shown as the number of spines per 20μm neuron segment (n = 50 per group from 5 mice per group). Comparisons of the average spine counts per animal was also unchanged between groups (not shown). **(D)** The ratio of the weighted tau (decay) of the extrasynaptic component (slow NMDA EPSC) to the synaptic (NMDA EPSC) component is not significantly different between both groups at 24h (n = 12 per group, 1 cell/animal). **(E)** Normalized percent charge transfer (%Q) is not significantly different between sham and HF-HI slices at 24h (n = 14 sham, n = 13 HF-HI, 1 cell/animal). **(F)** Input resistance was not significantly different for sham and HF-HI groups at 24 hours (n = 8 per group, 1 CA1 cell/mouse). **(G)** Capacitance, measured from the hyperpolarizing current clamp at 24 hours is not significantly different between sham and HF-HI cells (n = 8 per group, 1 CA1 cell/mouse). **(H)** Input resistance was not significantly different for sham and HF-HI groups at 1 month (n = 8 per group, 1 CA1 cell/mouse). **(I)** Capacitance, measured from the hyperpolarizing current clamp is not significantly different between sham and HF-HI cells and 1 month (n = 8 per group, 1 CA1 cell/mouse). Data shown as mean +/- SEM.

Supplemental Figure 6. Cortical transcriptomic signature 1m after HF-HI in wildtype mice and transcriptomic alterations induced by memantine pretreatment.

(A) Heatmap displaying the top 10 most significantly upregulated and downregulated genes in terms of Log2 fold change identified by RNA-seq. Heatmap of the Log2 fold change of the top 15 most significantly upregulated and downregulated genes in the Glutamatergic Synapse (CC) gene ontology category. **(B)** Functional enrichment of differentially expressed genes in HF-HI mice using gene sets from gene ontology (GO) displaying cellular components (CC) and biological processes (BP). **(C)** Heat map displaying the top 20 up and down regulated genes in the synapse (CC) category. Each gene is colored by Z (normalized standard deviations from the mean) for expression relative to the overall mean expression for that gene. Individual samples listed below each heat map. **(D)** Directionality dotplot of

saline and memantine treated HF-HI groups, obtained by functional enrichment of significantly differentially expressed genes using gene sets from Gene Ontology (GO) displaying biological processes (BP). **(E)** Heat map displaying all significantly differentiated learning or memory and **(F)** synaptic signaling biological process (BP) genes in saline and memantine treated mice. Color scale bar values represent Z-value (normalized standard deviations from the mean) for expression relative to the overall mean expression.

Supplemental Figure 7. Altered synaptic signatures are prominent features in post-mortem CTE brains and HF-HI mice.

Venn diagram demonstrating overlap of differentially expressed **(A)** biological process and **(B)** cellular component genes as identified by gene ontology functional enrichment of human CTE neuronal WGNCA clusters and mouse HF-HI transcripts. The top 12 overlapping pathways for both categories listed. **(C)** Heat map displaying the top differentiated genes in synaptic signaling BP categories. Hierarchical clustering shows separation by condition (CTE) v controls. Color scale bar values represent Z-value (normalized standard deviations from the mean) for expression relative to the overall mean expression. **(D)** Venn diagram demonstrating overlap of synaptic signaling (BP) genes in human CTE neuronal WGNCA clusters and mouse HF-HI transcripts. The 19 overlapping significantly changed genes listed.

Supplemental Figure 8. Post mortem human CTE transcriptomic network analysis.

Map of the most highly represented pathways changed by condition, generated using the enrichment map Cytoscape plug-in. Network analysis was performed using a false discovery rate (FDR) correction. BP, CC and KEGG are depicted for each gene set. Node size corresponds to the number of genes in each ontology term. Node color corresponds to which gene set each enrichment is derived from. Edges signify significant overlap between terms using a cutoff of 0.5 similarity coefficient, thicker edges indicate greater overlap between terms.

Supplemental Figure 1-Sloley & Main et al.

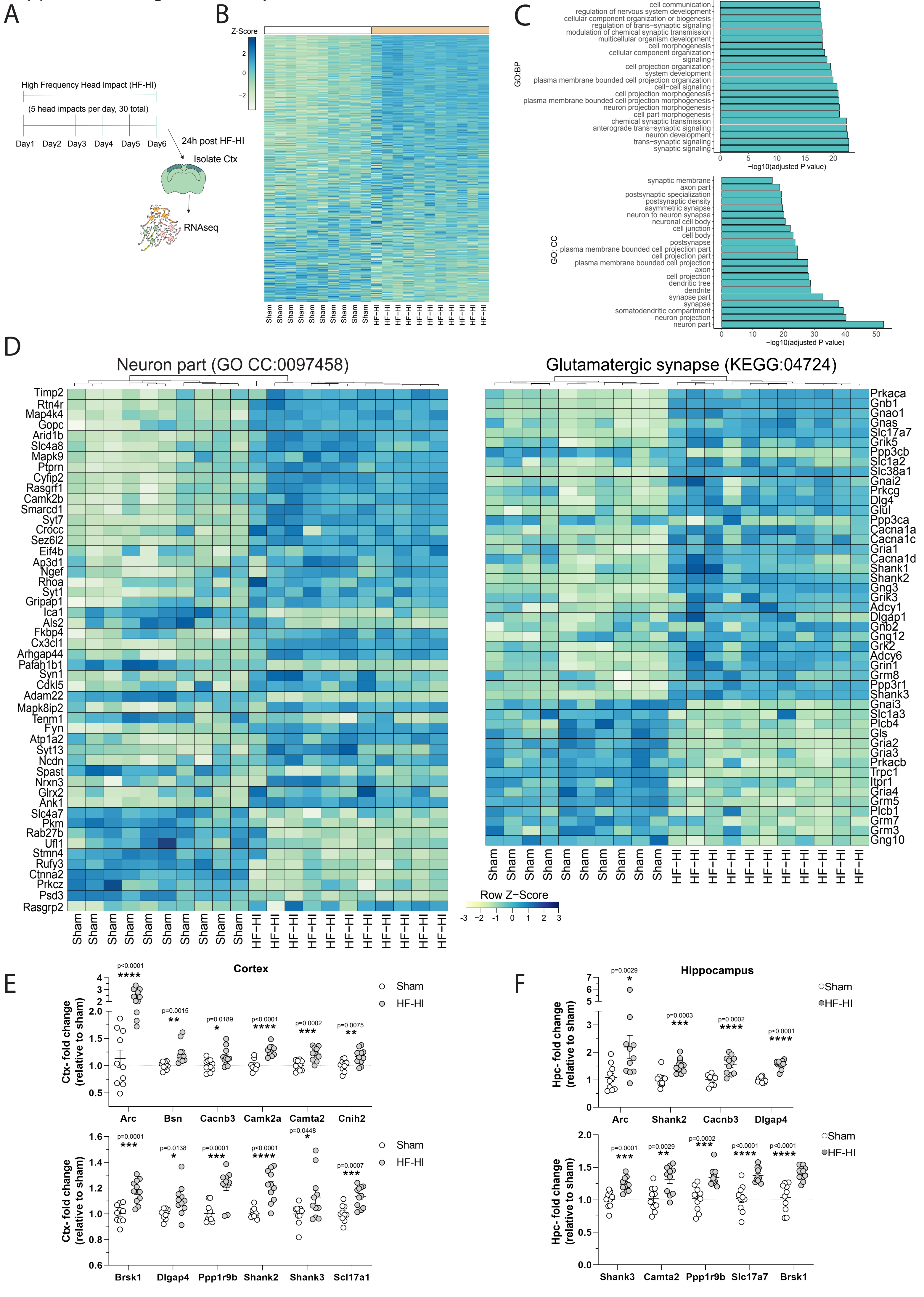

Supplemental Figure 2

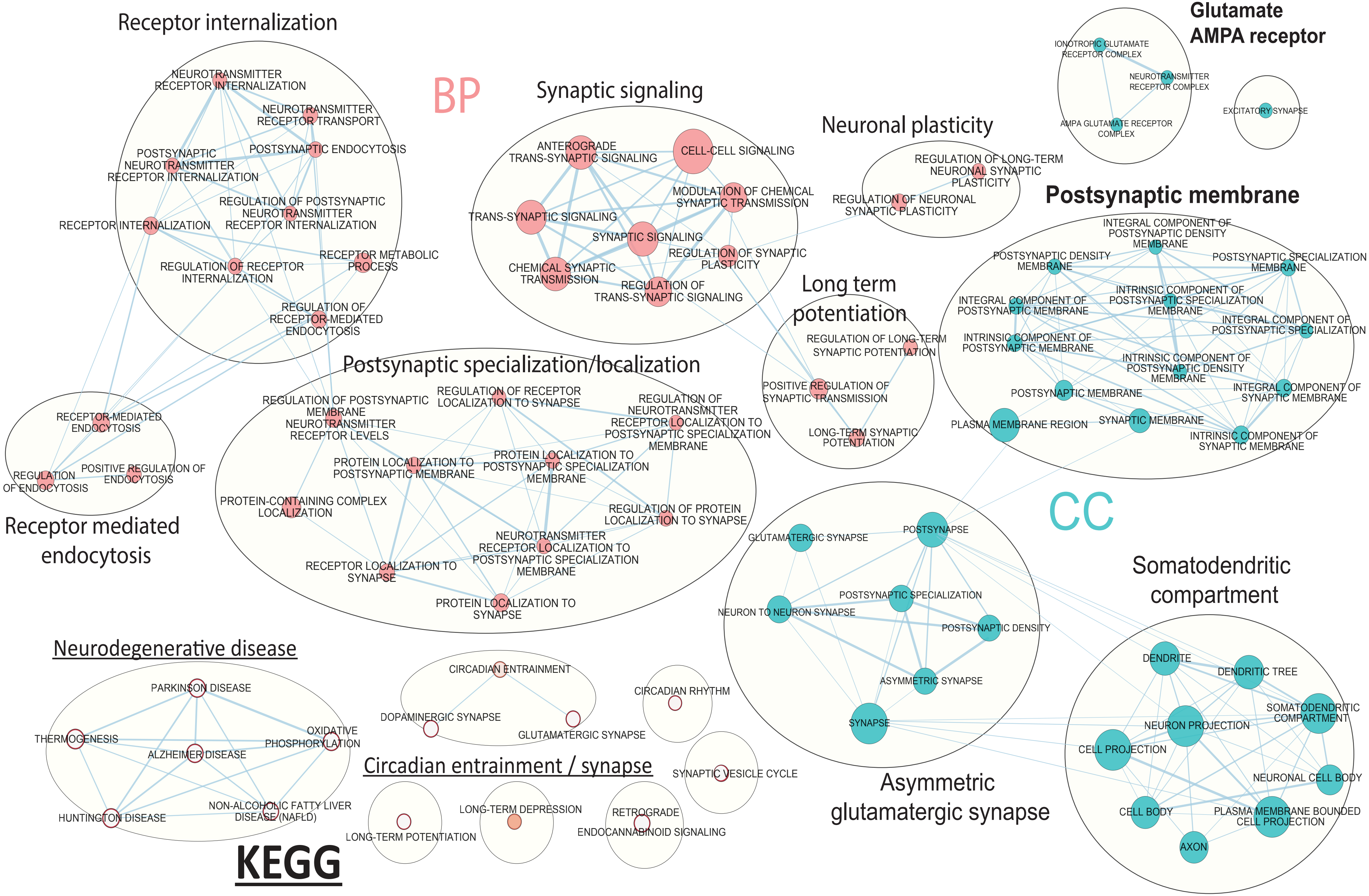

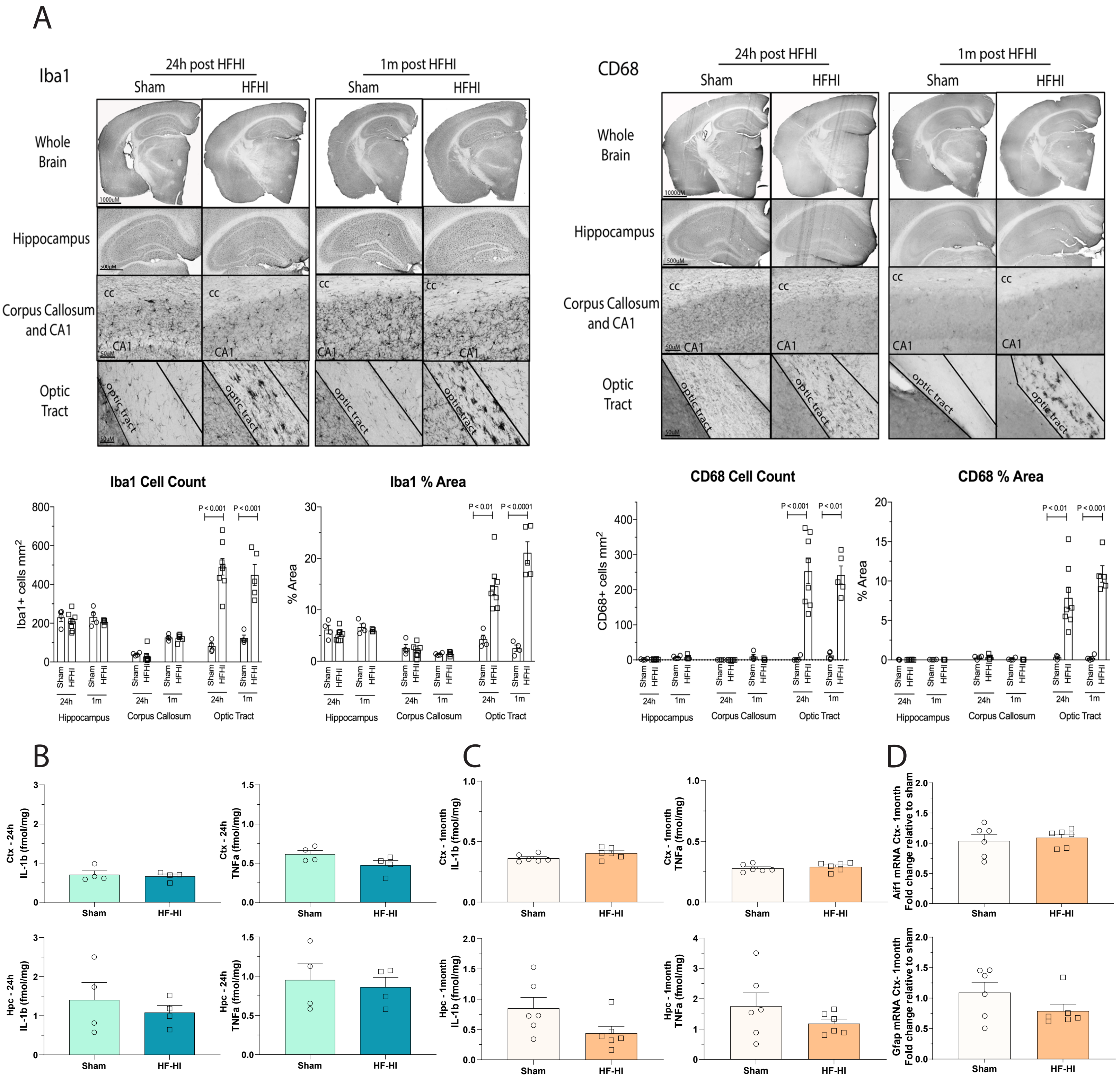
A
Iba1
24h post HFHI
1m post HFHI
Sham
HFHI
Whole Brain
Hippocampus
Corpus Callosum and CA1
Optic Tract
CD68
Iba1 Cell Count
Iba1 % Area
CD68 Cell Count
CD68 % Area
Iba1+ cells mm2
% Area
CD68+ cells mm2
P < 0.001
P < 0.01
P < 0.0001
Hippocampus
Corpus Callosum
Optic Tract
24h
1m
B
C
D
Ctx - 24h IL-1b (fmol/mg)
Ctx - 24h TNFa (fmol/mg)
Hpc - 24h IL-1b (fmol/mg)
Hpc - 24h TNFa (fmol/mg)
Ctx - 1month IL-1b (fmol/mg)
Ctx - 1month TNFa (fmol/mg)
Hpc - 1month IL-1b (fmol/mg)
Hpc - 1month TNFa (fmol/mg)
Aif1 mRNA Ctx- 1month Fold change relative to sham
Gfap mRNA Ctx- 1month Fold change relative to sham
Sham
HF-HI

Supplemental Figure 4

Sloley & Main et al.

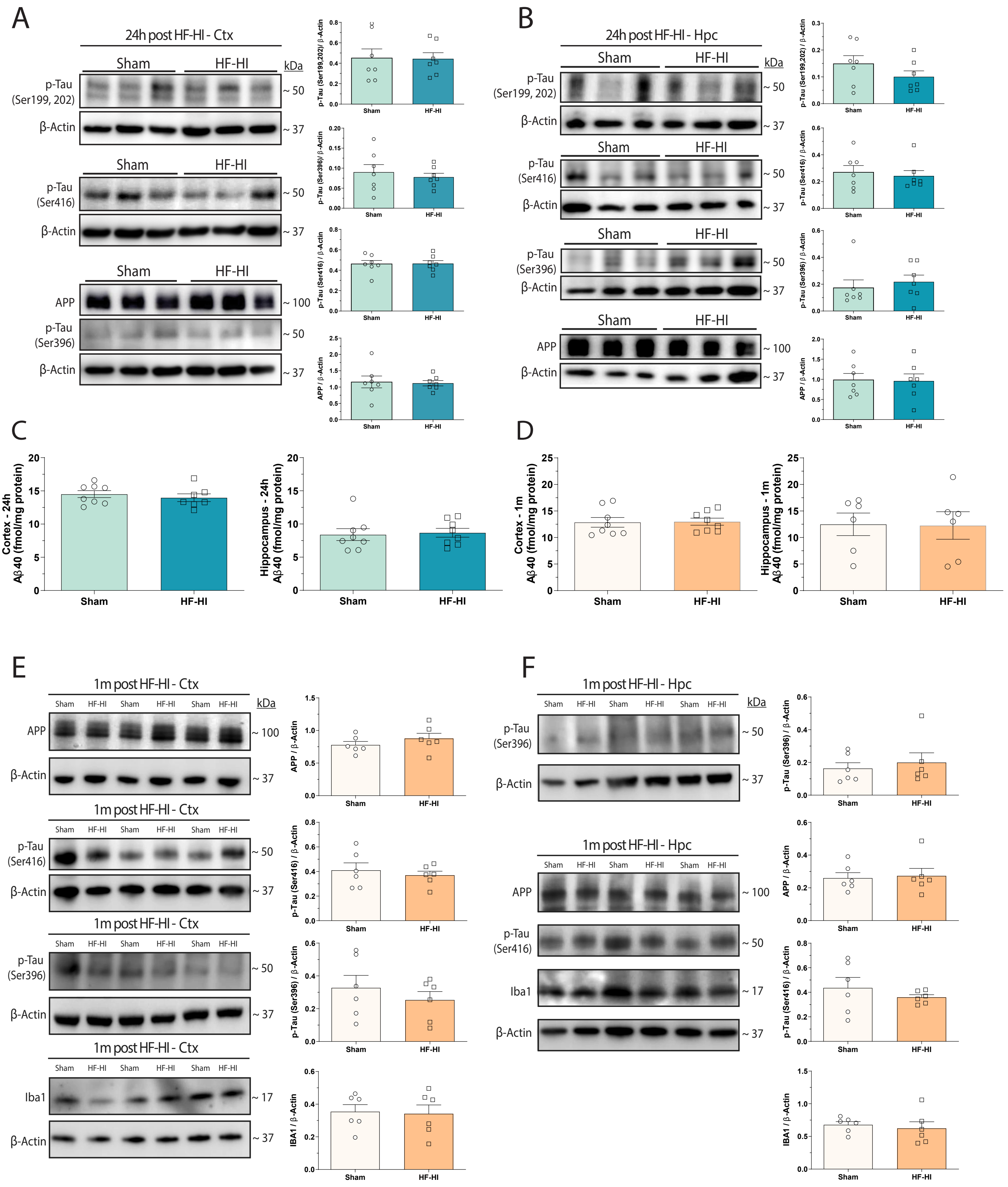

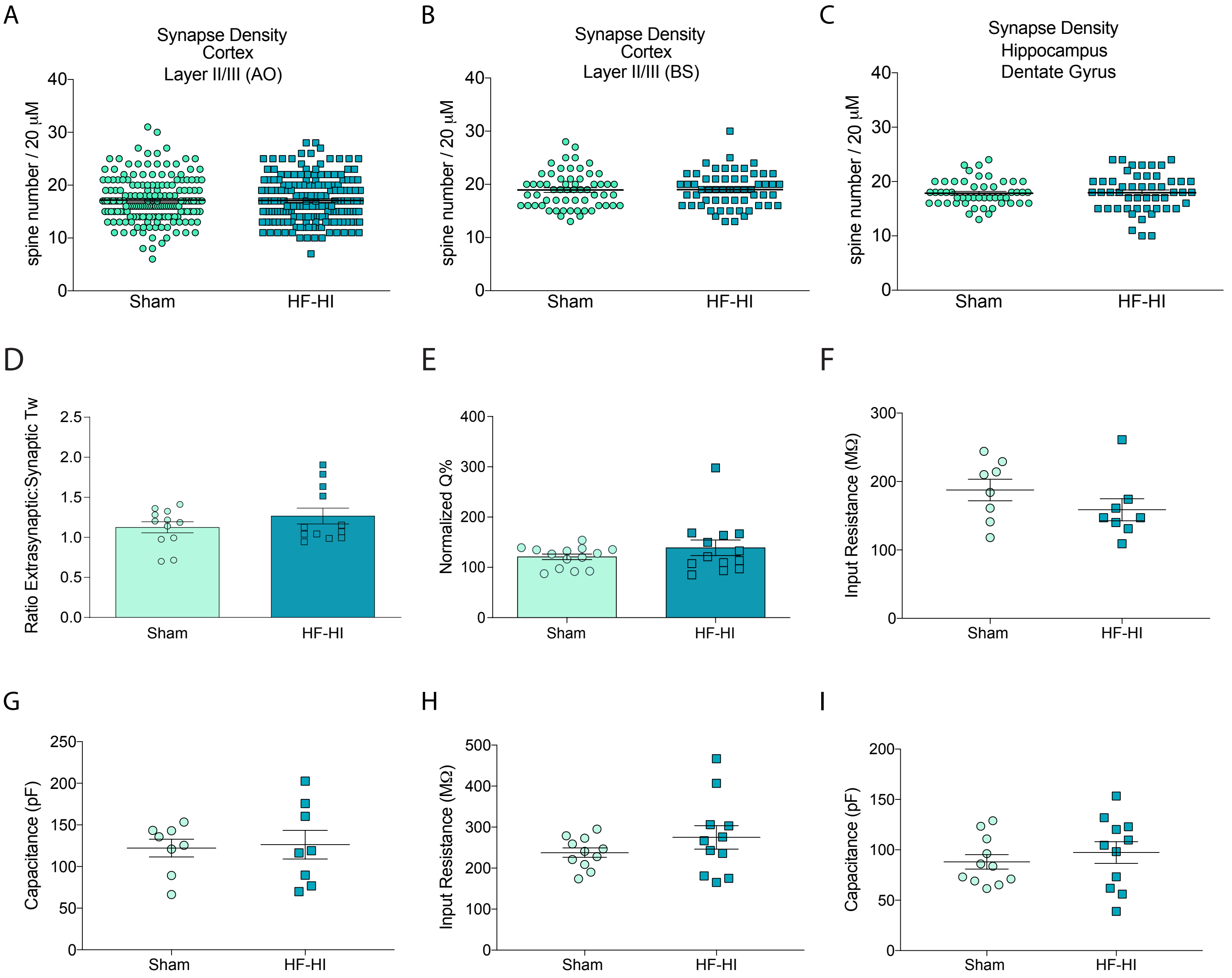
A
Synapse Density
Cortex
Layer II/III (AO)
spine number / 20 µM
Sham
HF-HI
B
Synapse Density
Cortex
Layer II/III (BS)
spine number / 20 µM
Sham
HF-HI
C
Synapse Density
Hippocampus
Dentate Gyrus
spine number / 20 µM
Sham
HF-HI
D
Ratio Extrasynaptic:Synaptic Tw
Sham
HF-HI
E
Normalized Q%
Sham
HF-HI
F
Input Resistance (MΩ)
Sham
HF-HI
G
Capacitance (pF)
Sham
HF-HI
H
Input Resistance (MΩ)
Sham
HF-HI
I
Capacitance (pF)
Sham
HF-HI

Supplemental Figure 6

Sloley & Main et al.

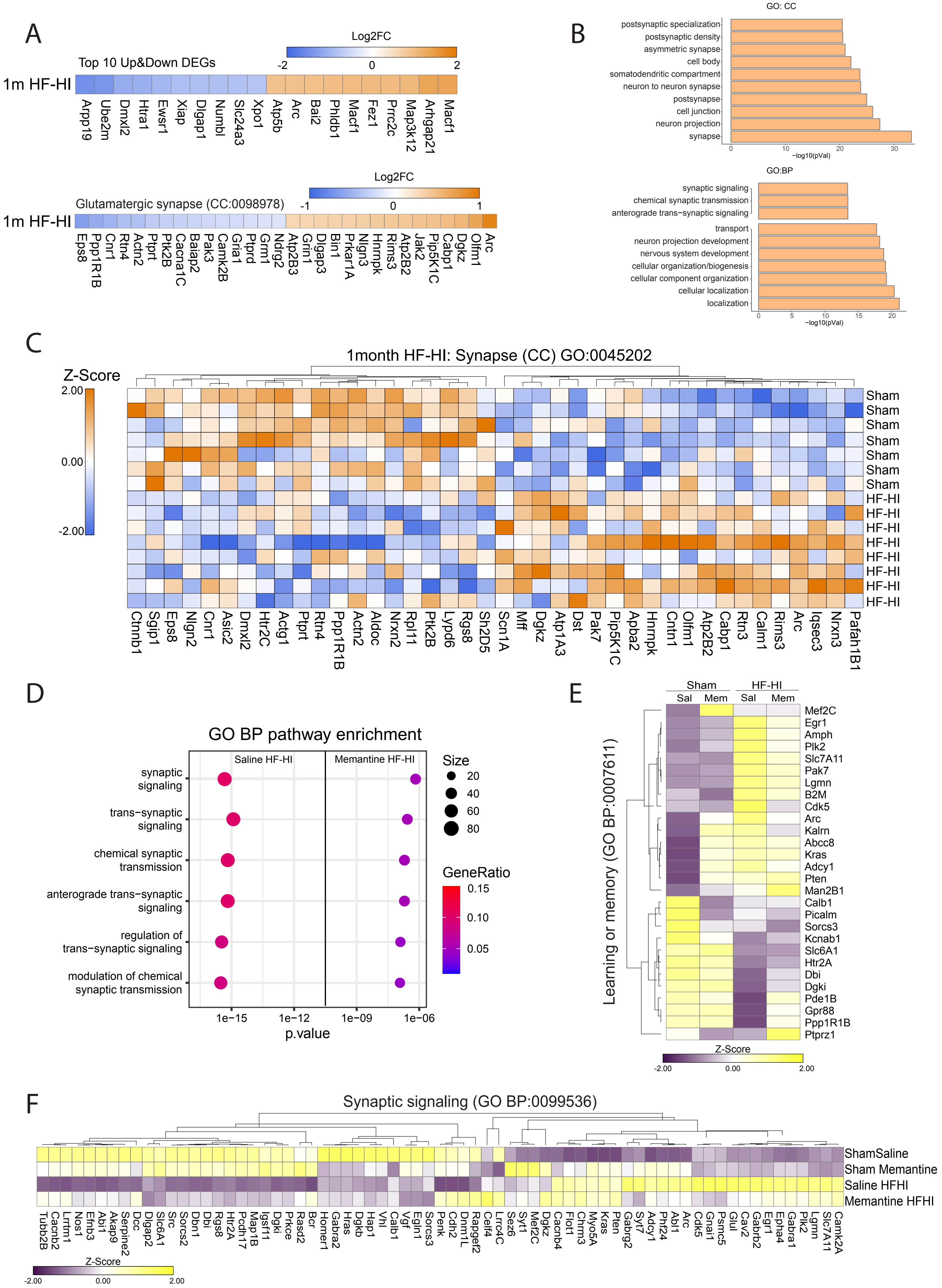

Supplemental Figure 7

Sloley & Main et al.

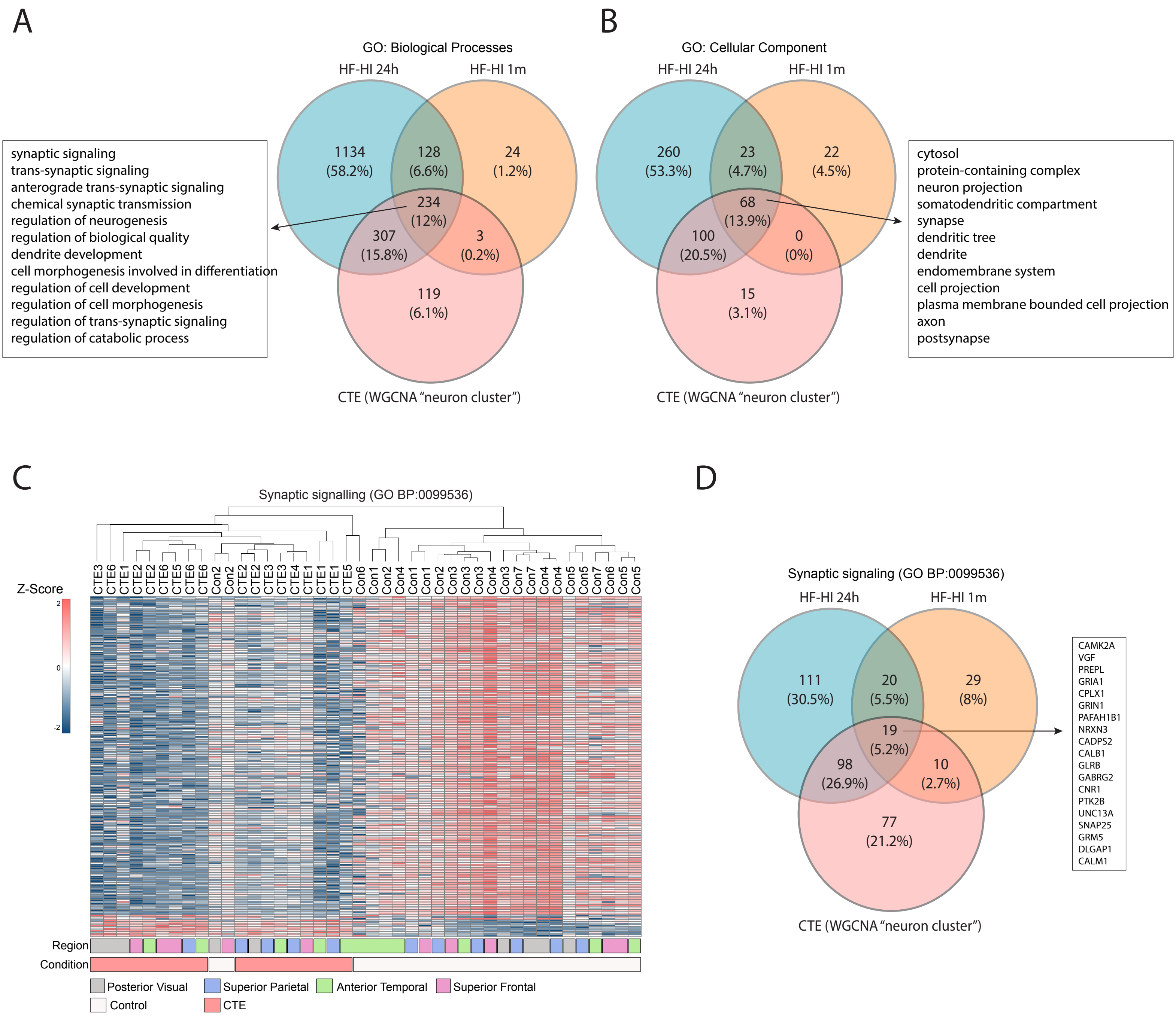

Supplemental Figure 8

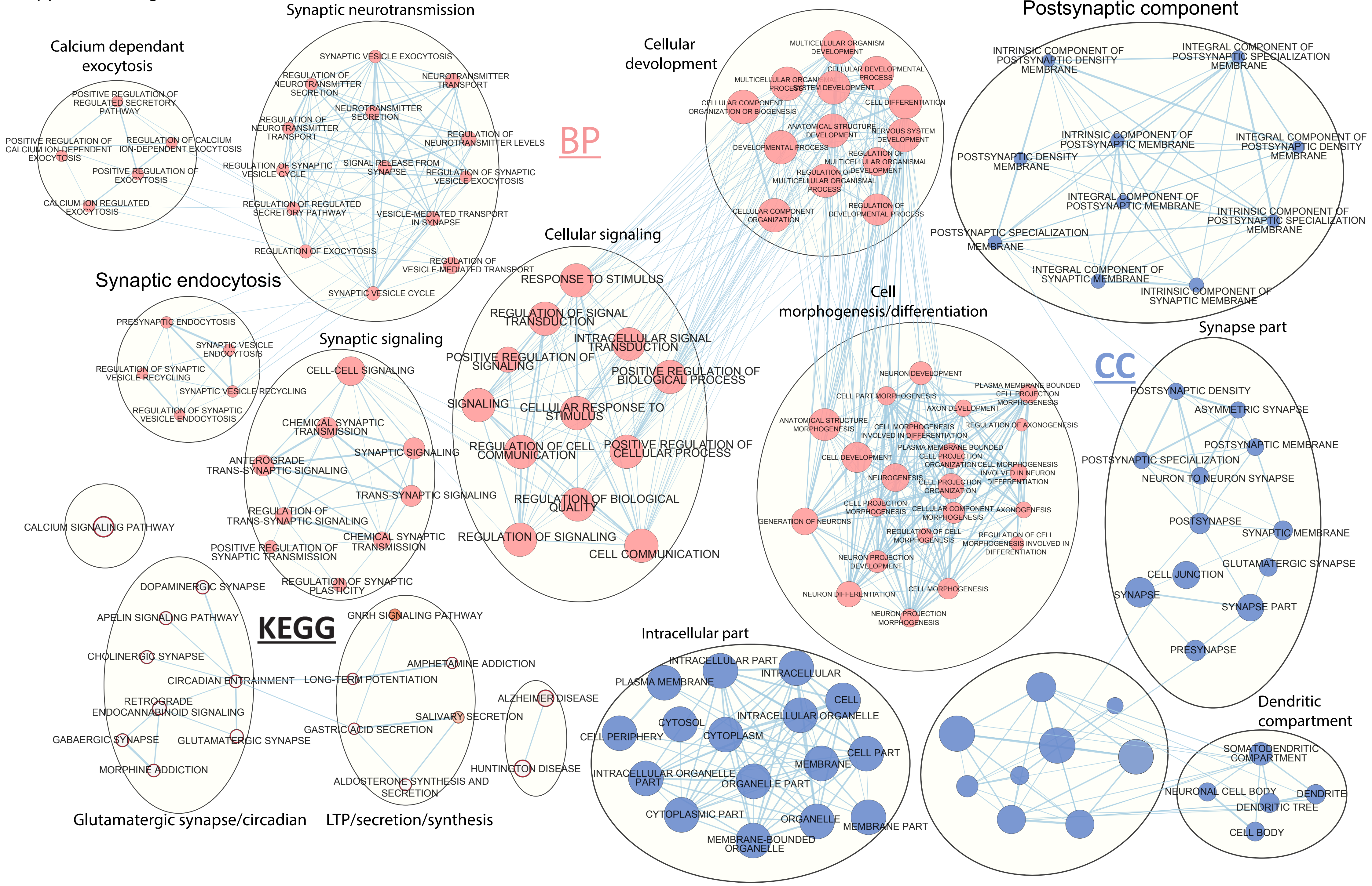

Supplement: Supplementary file 1 — Supplementary Information [file 41467_2021_22744_MOESM1_ESM.pdf]
