# Supplementary material for: High-frequency head impact causes chronic synaptic adaptation and long-term cognitive impairment in mice
Source: Nat Commun. 2021 May 10;12:2613. doi: 10.1038/s41467-021-22744-6 (PMC8110563; doi:10.1038/s41467-021-22744-6)
Supplement: Supplementary file 3 — Reporting Summary [file 41467_2021_22744_MOESM3_ESM.pdf]

nature research

Corresponding author(s): Mark P. Burns

Last updated by author(s): 03/12/2021

Reporting Summary

Nature Research wishes to improve the reproducibility of the work that we publish. This form provides structure for consistency and transparency in reporting. For further information on Nature Research policies, see our Editorial Policies and the Editorial Policy Checklist.

Statistics

For all statistical analyses, confirm that the following items are present in the figure legend, table legend, main text, or Methods section.

n/a	Confirmed	
☐	☒	The exact sample size (*n*) for each experimental group/condition, given as a discrete number and unit of measurement
☐	☒	A statement on whether measurements were taken from distinct samples or whether the same sample was measured repeatedly
☐	☒	The statistical test(s) used AND whether they are one- or two-sided<br>*Only common tests should be described solely by name; describe more complex techniques in the Methods section.*
☒	☐	A description of all covariates tested
☐	☒	A description of any assumptions or corrections, such as tests of normality and adjustment for multiple comparisons
☐	☒	A full description of the statistical parameters including central tendency (e.g. means) or other basic estimates (e.g. regression coefficient) AND variation (e.g. standard deviation) or associated estimates of uncertainty (e.g. confidence intervals)
☐	☒	For null hypothesis testing, the test statistic (e.g. *F*, *t*, *r*) with confidence intervals, effect sizes, degrees of freedom and *P* value noted<br>*Give P values as exact values whenever suitable.*
☒	☐	For Bayesian analysis, information on the choice of priors and Markov chain Monte Carlo settings
☐	☒	For hierarchical and complex designs, identification of the appropriate level for tests and full reporting of outcomes
☒	☐	Estimates of effect sizes (e.g. Cohen's *d*, Pearson's *r*), indicating how they were calculated

Our web collection on statistics for biologists contains articles on many of the points above.

Software and code

Policy information about availability of computer code

Data collection	Clampfit v10.7 (Molecular Devices) was used for data analysis of all electrophysiology studies. ANY-Maze v6.1 (Stoelting Co) was used to track and record data in MWM and Barnes Maze behavioral experiments. Western imaging was conducted with an Amersham 600 (GE Healthcare). Histology images acquired on an Olympus XB51 microscope equipped with an Olympus DP72 camera. RT-QPCR was performed using the 7900ht-Fast apparatus (Applied Biosystems). Graphical illustrations were made using BioRender.

Data analysis	Graph Pad Prism 8 was used to represent data in graphs and for the statistical analyses of the data. ImageJ software(v 1.47, https://imagej.nih.gov/ij/download.html) was used to outline and measure areas for histology and western blot imaging analysis. Clampfit v10.7 - Molecular Devices was used for data analysis of all electrophysiology studies. ANY-maze™ tracking system v6.1 (Stoelting Co.) was used to track mice for MWM and Barnes maze behavioral experiments. Heat maps displaying time spent in apparatus was generated within ANYmaze ™. RNA sequencing raw reads (FASTQ files) were aligned to the mouse reference genome (mm10) using STAR and expression counts per transcript quantified using eXpress. DESeq2 (version 3.6.3) was used to normalize the raw counts based on read depth and perform differential expression analysis. Evaluation of Gene Ontology term enrichments for both HF-HI and CTE studies was performed using gProfileR within R and from the gProfiler database (https://biit.cs.ut.ee/gprofiler/gost). Following DESeq2 analysis and generation of variance stabilized contrasts with Log2 fold change data, pca, bar, volcano and dot plots were generated from datasets using ggplot2 [https://ggplot2.tidyverse.org] and enhanced volcano plot packages [https://github.com/kevinblighe/EnhancedVolcano]. Heatmaps were generated using the pheatmap R package [https://github.com/raivokolde/pheatmap], and Morpheus software [https://software.broadinstitute.org/morpheus]. The GWAS Catalog was used to find genes associated with neurodegenerative or cognitive/behavioral traits (https://www.ebi.ac.uk/gwas/home), and the circos plot including these data was generated using the circlize R package. Cytoscape v3.8.0 (https://cytoscape.org/) was used to visualize protein interaction networks from the gProfiler database. gSDS 2.4 software (Applied Biosystems) was used to generate threshold cycle (Ct) values. Microsoft Excel was used to compile data.

For manuscripts utilizing custom algorithms or software that are central to the research but not yet described in published literature, software must be made available to editors and reviewers. We strongly encourage code deposition in a community repository (e.g. GitHub). See the Nature Research guidelines for submitting code & software for further information.

Data

Policy information about availability of data

All manuscripts must include a data availability statement. This statement should provide the following information, where applicable:

- Accession codes, unique identifiers, or web links for publicly available datasets
- A list of figures that have associated raw data
- A description of any restrictions on data availability

The source data and statistical analysis for all figures are provided with the paper in Microsoft Excel format. The source data underlying Figures 2,3,6,7,8 and Supplementary Figures 1-3,6 are provided as an excel data source file with all statistical analysis performed. Raw and processed sequencing data (all remaining figures) are provided as supplemental tables of the manuscript and can be accessed through the Gene Expression Omnibus (GEO) accession code GSE165760; https://www.ncbi.nlm.nih.gov/geo/query/acc.cgi?acc=GSE165760. Human CTE RNAseq data was extracted from European Nucleotide Archive database (ERP015139): https://www.ebi.ac.uk/ena/browser/view/PRJEB13579

Field-specific reporting

Please select the one below that is the best fit for your research. If you are not sure, read the appropriate sections before making your selection.

☒ Life sciences ☐ Behavioural & social sciences ☐ Ecological, evolutionary & environmental sciences

For a reference copy of the document with all sections, see nature.com/documents/nr-reporting-summary-flat.pdf

Life sciences study design

All studies must disclose on these points even when the disclosure is negative.

Sample size	Sample sizes for each experiment were calculated using G*Power v3.1,49 and based on the standard power calculations where effect size was set at 25%, a-error probability at 0.05, and power at 80%. All sample sizes are indicated in figure legends and are outlined in supplementary tables providing the raw data of the group means for each experiment, exact n numbers used in each data panel presented, t-values, degrees of freedom (df), f-values, exact p-values and the analysis used for all data presented in the manuscript.
Data exclusions	No data were excluded for analysis
Replication	The following data in the manuscript were successfully repeated one time with independent cohorts: Barnes Maze, Water Maze, T-maze, mouse RNAseq. The key-finding data elements including the 24h transcriptome data and the AMPA/NMDA ratio data were also successfully replicated in the memantine study, and that data is included in the manuscript.
Randomization	All animals were randomized prior to the beginning of each study.
Blinding	Study investigators were blinded to head impact or sham condition, and to treatment group.

Reporting for specific materials, systems and methods

We require information from authors about some types of materials, experimental systems and methods used in many studies. Here, indicate whether each material, system or method listed is relevant to your study. If you are not sure if a list item applies to your research, read the appropriate section before selecting a response.

Materials & experimental systems

n/a	Involved in the study
☐	☒ Antibodies
☒	☐ Eukaryotic cell lines
☒	☐ Palaeontology and archaeology
☐	☒ Animals and other organisms
☒	☐ Human research participants
☒	☐ Clinical data
☒	☐ Dual use research of concern

Methods

n/a	Involved in the study
☒	☐ ChIP-seq
☒	☐ Flow cytometry
☒	☐ MRI-based neuroimaging

Antibodies

Antibodies used

anti-APP (36-6900, Rabbit IgG; Invitrogen, 1:1000), anti-CD68 (MCA1957T, mouse IgG; Serotec, 1:1000), anti-beta Actin (A5441, mouse IgG; Sigma-Aldrich, 1:5000), anti-Ibal (019-19741, rabbit IgG; Waka, 1:1000), anti- p-Tau Ser199, Ser202 (44-768G, Rabbit IgG,Invitrogen, 1:1000), anti- p-Tau Ser396, PHF13 (9632, Rabbit IgG, Cell Signaling, 1:1000), anti- p-Tau Ser416 (15013, Rabbit IgG, Cell Signaling, 1:1000), goat anti-mouse (115-035-003, RRID: AB_10015289, JacksonImmunoResearch, 1:1000), goat anti-rabbit (115-035-003, RRID: AB_2313567, JacksonImmunoResearch, 1:1000). Biotinylated goat anti-rabbit (1:2000, Vector, BA-1000) or anti-mouse secondary antibodies (1:2000, Vector, BA-9200)

Validation

All are well established antibodies that have been validated in multiple labs over many years. All antibodies were validated by the manufacturer. anti-APP (36-6900, Invitrogen): 13 publications, Usage: WB, ELISA, IHC, ICC, IHC(P), IF. anti-CD68 (MCA1957T, Clone FAll, Serotec,): 648 publications, Usage: WB, IHC, IF, IP, FC. anti-beta Actin (A5441, Clone AC-15, Sigma-Aldrich): 7057 publications, Usage: WB, IHC, ELISA, IF. anti-Ibal (019-19741, Waka,): 340 publications, Usage: WB, IHC. anti- p-Tau Ser199, Ser202 (44-768G, Invitrogen): 17 publications, Usage: WB, IHC, IHC(P), ELISA. anti- p-Tau Ser396, PHF13 (9632, Cell Signaling): 29 publications, Usage: WB. anti- p-Tau Ser416 (15013, Cell Signaling): 5 publications, Usage: WB, IP, IHC. (BA-1000, vector): 6487 publications, Usage: WB, IHC, ELISA. (BA-9200, vector): 1827 publications, Usage: WB, IHC, ELISA.

Animals and other organisms

Policy information about studies involving animals; ARRIVE guidelines recommended for reporting animal research

Laboratory animals

Six- to twelve- week old male C57Bl/6J obtained from Jackson Laboratories were used for experiments. Mice were group housed under a 12 h light/dark cycle with ad libitum access to food and water. Rooms were maintained at 18-24C and 40-60% humidity. 6 week old mice were used for electrophysiolgy studies. All other mice were 10-12 weeks of age. Mice were matched for age before random assigned to experimental groups.

Wild animals

Study did not involve wild animals

Field-collected samples

Study did not involve field collected samples

Ethics oversight

All procedures were performed in accordance with protocols approved by the Georgetown University Animal Care and Use Committee.

Note that full information on the approval of the study protocol must also be provided in the manuscript.
